# Supplementary material for: Determination of Asphaltene Critical Nanoaggregate Concentration Region Using Ultrasound Velocity Measurements
Source: Sci Rep. 2017 Nov 23;7:16125. doi: 10.1038/s41598-017-16294-5 (PMC5700960; doi:10.1038/s41598-017-16294-5)
Supplement: Supplementary file 1 — Supplementary Information [file 41598_2017_16294_MOESM1_ESM.pdf]

Determination of Asphaltene Critical Nanoaggregate Concentration Region Using Ultrasound Velocity Measurements. Supplementary Information.

Aleksandra Svalova[1,*], Nicholas G Parker[2], Malcolm JW Povey[3], and Geoffrey D Abbott[1]

[1]Newcastle University, School of Natural and Environmental Sciences, Newcastle upon Tyne, NE1 7RU, United Kingdom
[2]Newcastle University, School of Mathematics, Statistics and Physics, Newcastle upon Tyne, NE1 7RU, United Kingdom
[3]University of Leeds, School of Food Science and Nutrition, Leeds, LS2 9JT, Leeds, United Kingdom
*a.svalova@newcastle.ac.uk

Constrained optimization with penalty functions

What follows describes the use of constrained optimisation for critical nanoaggregate region (CNR) estimation. Constrained optimisation (CO) is a statistical method allowing to select an optimal model from a set of several possibilities[1,2]. Suppose there exists a number of models that show similar performance quality based on some statistic, called the objective function. To select the optimal model, a set of rules/constraints is applied whose violation results in penalising of the objective function. Here, CO is applied to linear regression selection by penalising the coefficient of determination R^2 (the objective function). The advantage of using R^2 over the mean squared error is due to the use of both, the variability explained by the regression and the total variation in the data. Additionally, R^2 is a more popular regression performance measure in applied statistical fields.
In an optimisation problem the objective function is re-evaluated by penalties $g(\cdot)$, given the observance or violation of user-defined constraints. This produces the formalism of a penalised objective function, R_p^2 in our case, defined as

$$R_p^2 = R^2 - \sum_{i=1}^{n} C_i \delta_i g_i(x), \quad i = 1, 2, \ldots, n, \quad n - \text{number of constraints}, \tag{1}$$

$$\delta_i = \begin{cases} 1 & \text{if constraint i is violated,} \\ 0 & \text{if constraint i is met.} \end{cases}$$

The constant C_i represents the penalty magnitude for the violation of constraint i which may be set to increase with the magnitude of constraint violation. The selected CNR boundaries were generally independent of changes in C_i and the final constants were chosen to satisfy $g_i(\cdot)C_i < 1$ so that the interpretation of R_p^2 remained intuitive.
We analysed all possible non-overlapping two-regression combinations by varying the number of points in the monomeric/aggregated regressions. Constraints $g_i(\cdot)$ allowed to combine a priori knowledge about the behaviour underlying asphaltene aggregation process with the velocity measurements. Three constraints were defined as follows.

1. Initial screening of velocity data illustrated that models with a lower number of points led to higher R^2 values regardless of whether the CNR was approached or not. We penalised all R^2 by $g_1(i) = 1/\sqrt{i}$ where i is the number of points included in a regression.

2. When fitting a linear regression through monomeric/aggregated regions or the CNR, the residual distributions will be different due to varying sources of uncertainty, e.g. instrument measurement error vs a different underlying model. Therefore, $g_2(i)$ is as follows:

   (a) Fit a linear regression M_i to the first i velocity-concentration measurements.

   (b) Obtain the residuals of M_i, calculate their 95% confidence interval I.

   (c) Estimate the value m_{i+1} of the point$_{i+1}$ using M_i.

   (d) Obtain the resudual $e_{i+1} = m_{i+1} - velocity_{i+1}$. If e_{i+1} lies outside I, penalise R^2 by $g_2(i) = e_{i+1}$.

3. Some measurements will cause a change in regression slope without producing a large outlier which may also be a sign of entering the CNR. Therefore, $g_3(i)$ is the difference between regression slopes fitted to the first i and $i+1$ points.

We tested this model on pure CTAB mixtures and obtained the CMC estimations that matched those in our ultrasonic velocity measurements.

References

1. Bertsekas, D. P. *Constrained optimization and Lagrange multiplier methods* (Athena Scientific, Massachussets, 1982).

2. Smith, A. E. & Coit, D. W. Constraint-handling techniques - penalty functions. In Baeck, T., Fogel, D. & Michalewicz, Z. (eds.) *Handbook of Evolutionary Computation* (Institute of Physics Publishing and Oxford University Press, Bristol, 1997).

3. Brincat, D. & Abbott, G. D. Some aspects of the molecular biogeochemistry of massive and laminated rocks from naples beach section (santa barbara-ventura basin). In Isaacs, C. M. & Rüllkötter, J. (eds.) *The Monterey formation: from rocks to molecules*, 140–149 (Colombia University Press, New York, 2001).

4. Abbott, G. D., Lewis, C. & Maxwell, J. R. Laboratory models for aromatization and isomerization of hydrocarbons in sedimentary basins. *Nat.* **318**, 651–653 (1985).

5. Abbott, G. D., Lewis, C. A. & Maxwell, J. R. The kinetics of specific organic reactions in the zone of catalysis. *Philos. Transactions Royal Soc. Lond. A* **315**, 107–122 (1985).

Supplementary figures

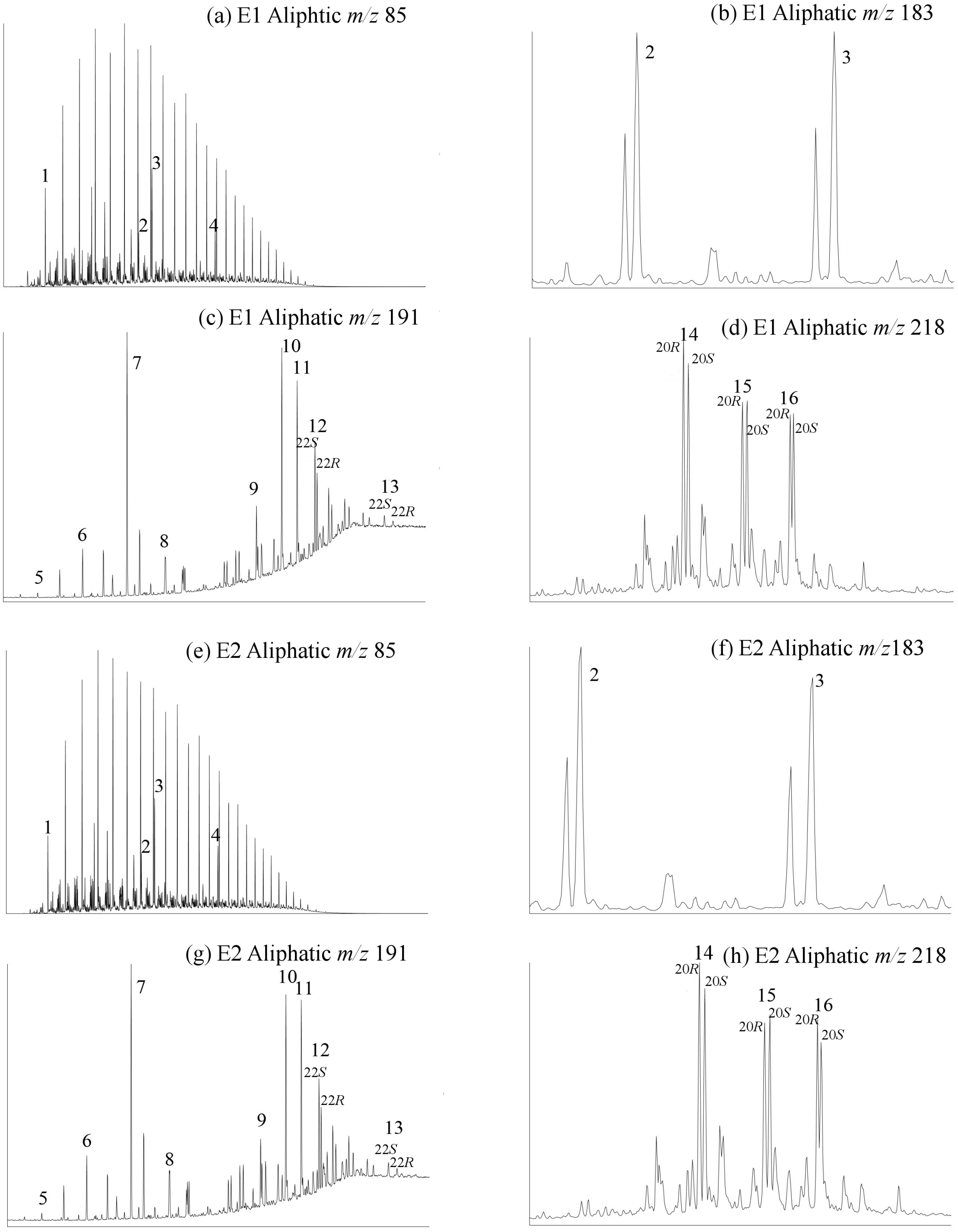

Figure S1. Partial chromatograms (retention time (min) versus intensity) supplementing biodegradation analysis. Compounds are listed in Table 1.

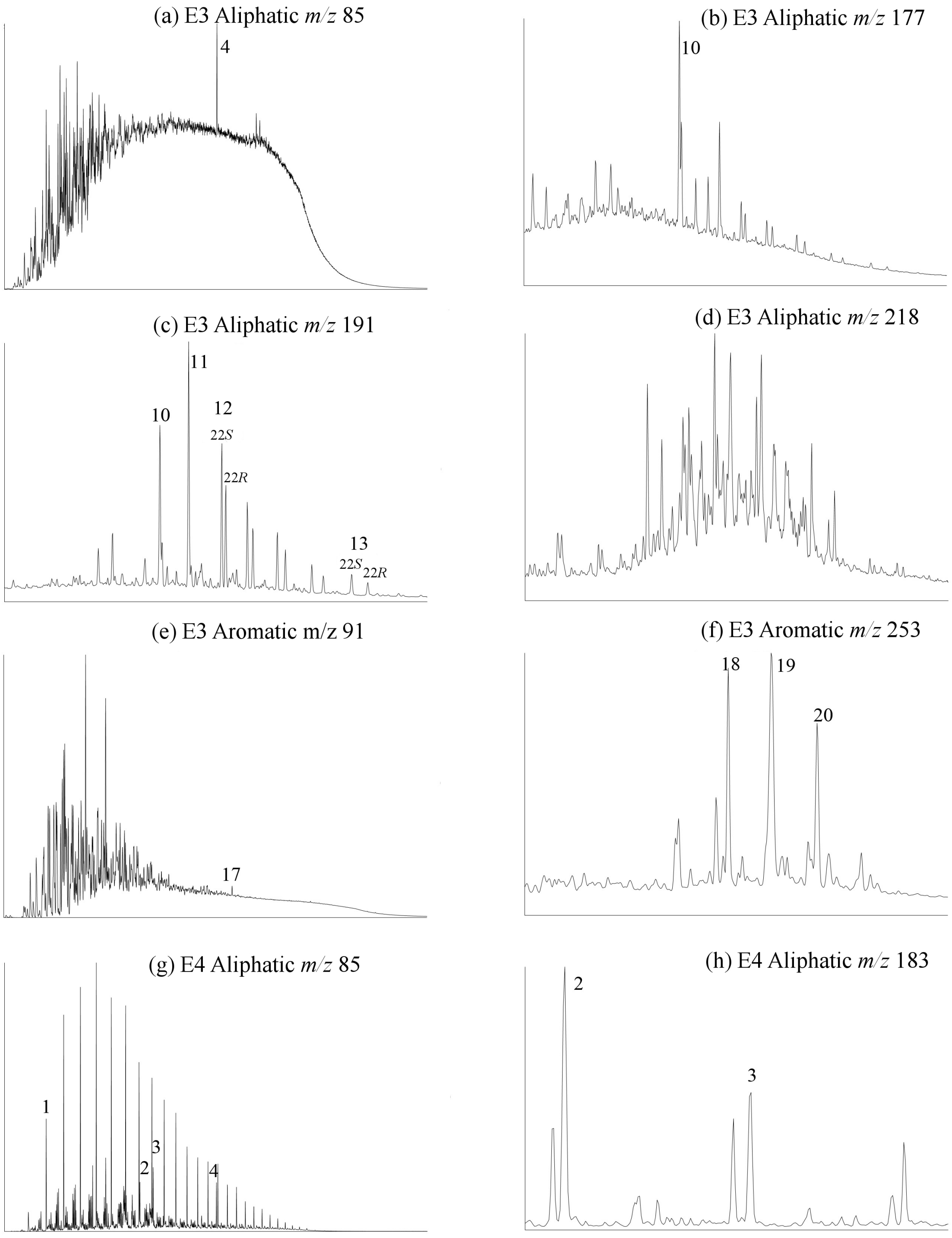

Figure S2. Partial chromatograms (retention time (min) versus intensity) supplementing biodegradation analysis. Compounds are listed in Table 1.

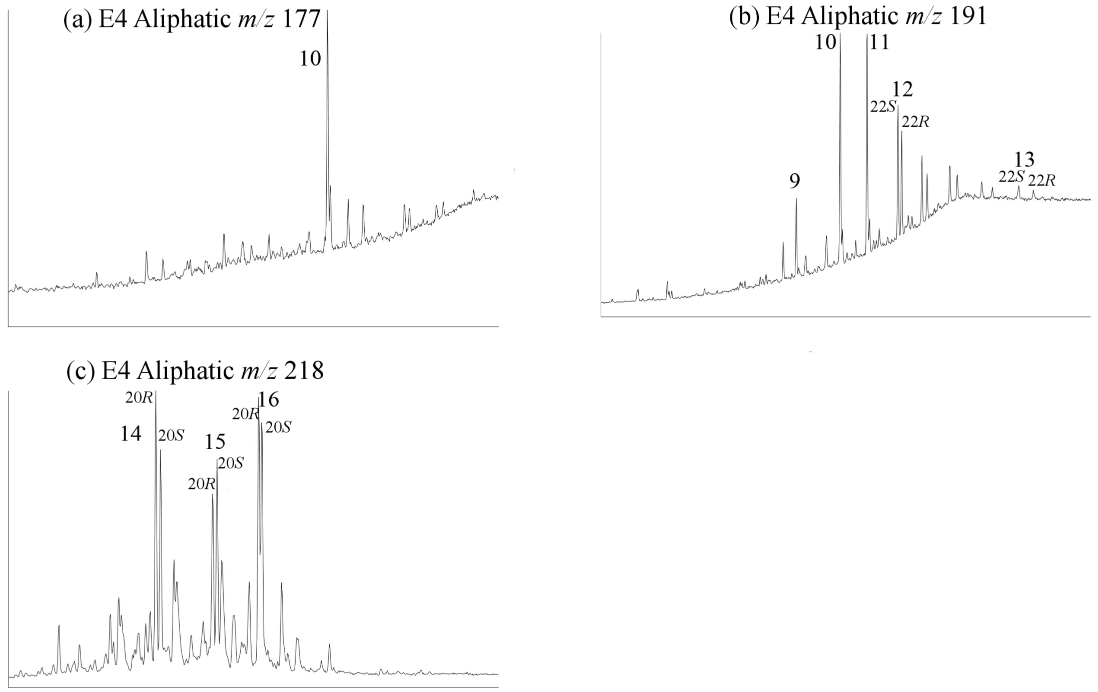

Figure S3. Partial chromatograms (retention time (min) versus intensity) supplementing biodegradation analysis. Compounds are listed in Table 1.

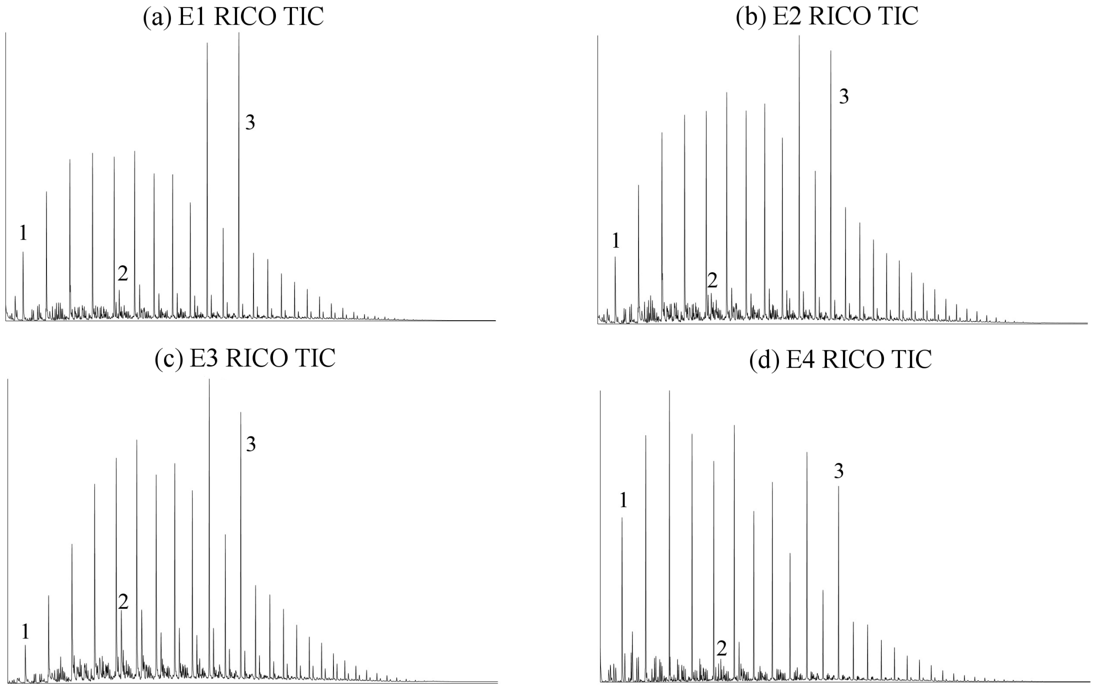

Figure S4. Total ion chromatograms (TIC; retention time (min) versus intensity) supplementing RICO analysis. Compounds are listed in Table 2.

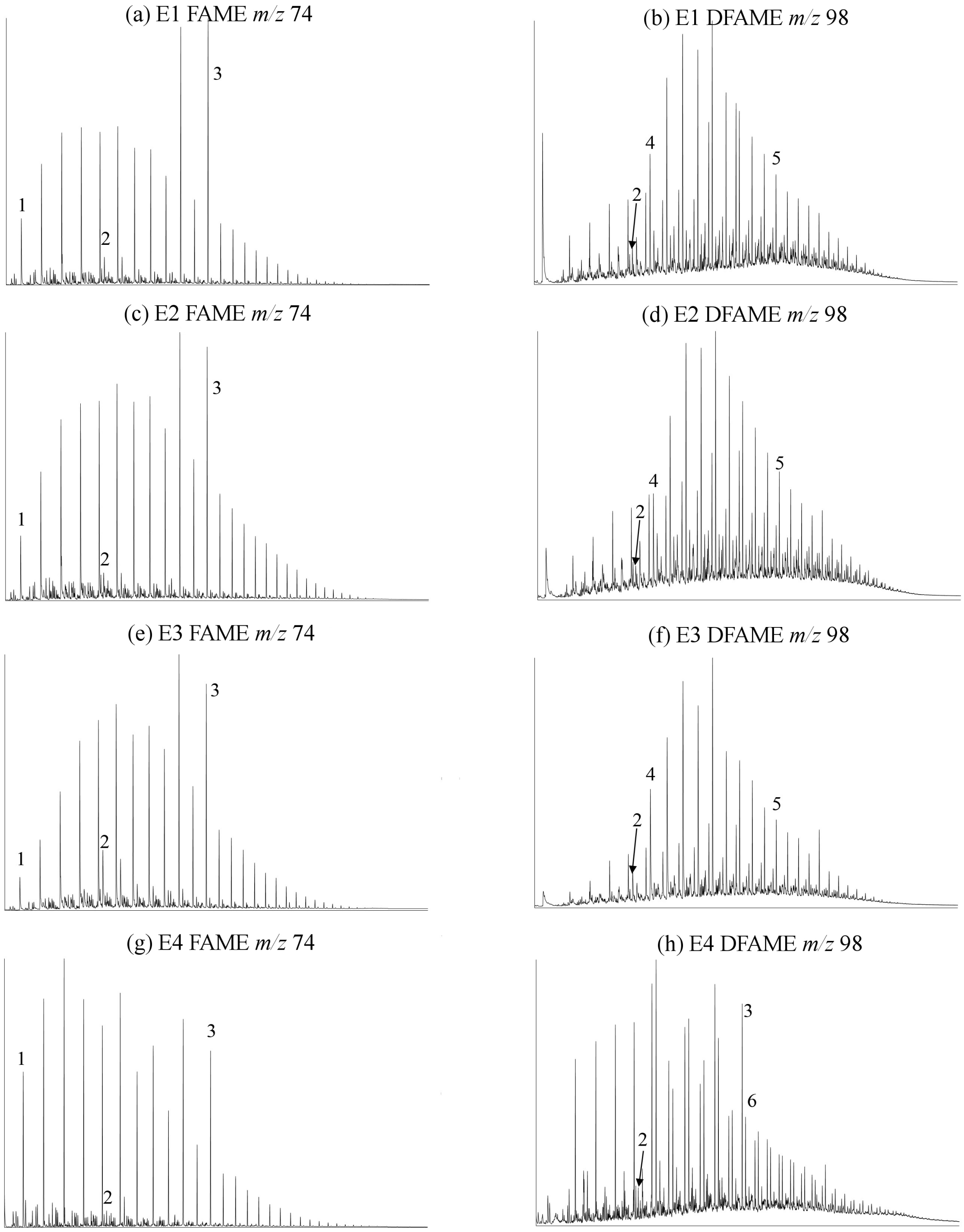

Figure S5. Partial chromatograms (retention time (min) versus intensity) supplementing RICO analysis. Compounds are listed in Table 2.

Number	Name
1	*n*-undecane
2	2,6,10,14-tetramethylpentadecane (pristane)
3	2,6,10,14-tetramethylxehadecane (phytane)
4	heptadecylcyclohexane
5[3]	(C_{19}) tricyclic terpane
6[3]	(C_{21}) tricyclic terpane
7[3]	(C_{23}) tricyclic terpane
8[3]	(C_{25}) tricyclic terpane
9[3]	(C_{27}) 17α-22,29,30-*trinor*-hopane
10[3]	(C_{29}) $17\alpha,21\beta$-*nor*-hopane
11[3]	(C_{30}) $17\alpha,21\beta$-*nor*-hopane
12[3]	(C_{31}) $17\alpha,21\beta$-*homo*-hopane 22*S* and 22*R* epimers
13[3]	(C_{35}) $17\alpha,21\beta$-*pentakishomo*-hopane 22*S* and 22*R* epimers
14[3]	(C_{27}) $5\alpha,14\beta,17\beta$-cholestane 20*R* and 20*S* epimers
15[3]	(C_{28}) $5\alpha,14\beta,17\beta$-24-methylcholestane 20*R* and 20*S* epimers
16[3]	(C_{29}) $5\alpha,14\beta,17\beta$-24-ethylcholestane 20*R* and 20*S* epimers
17	1,4-diphenylbenzene (*p*-terphenyl)
18[4,5]	(C_{27}+C_{28}) C-ring monoaromatic steroid
19[4,5]	(C_{27}+C_{28}+C_{29}) C-ring monoaromatic steroid
20[4,5]	(C_{29}) C-ring monoaromatic steroid

Table 1. Compound table for Figures S1-S3.

Number	Name
1	*n*-heptanoic acid methyl ester
2	α,ω-*di*-*n*-octanedioic acid *bis*-methyl ester
3	*n*-octadecanoic acid methyl ester
4	α,ω-*di*-*n*-nonanedioic acid *bis*-methyl ester
5	α,ω-*di*-*n*-octadecanedioic acid *bis*-methyl ester
6	α,ω-*di*-*n*-pentadecanedioic acid *bis*-methyl ester

Table 2. Compound table for Figures S4 and S5.
